# Discovery of Mating in the Major African Livestock Pathogen *Trypanosoma congolense*

**DOI:** 10.1371/journal.pone.0005564

**Published:** 2009-05-15

**Authors:** Liam J. Morrison, Alison Tweedie, Alana Black, Gina L. Pinchbeck, Robert M. Christley, Andreas Schoenefeld, Christiane Hertz-Fowler, Annette MacLeod, C. Michael R. Turner, Andy Tait

**Affiliations:** 1 Wellcome Centre for Molecular Parasitology, University of Glasgow, Glasgow Biomedical Research Centre, Glasgow, United Kingdom; 2 Faculty of Biomedical and Life Sciences, University of Glasgow, Glasgow Biomedical Research Centre, Glasgow, United Kingdom; 3 Faculty of Veterinary Science, University of Liverpool, Leahurst, Neston, United Kingdom; 4 International Trypanotolerance Centre, Banjul, The Gambia; 5 Sanger Institute, Wellcome Trust Genome Campus, Hinxton, Cambridge, United Kingdom; BMSI-A*STAR, Singapore

## Abstract

The protozoan parasite, *Trypanosoma congolense*, is one of the most economically important pathogens of livestock in Africa and, through its impact on cattle health and productivity, has a significant effect on human health and well being. Despite the importance of this parasite our knowledge of some of the fundamental biological processes is limited. For example, it is unknown whether mating takes place. In this paper we have taken a population genetics based approach to address this question. The availability of genome sequence of the parasite allowed us to identify polymorphic microsatellite markers, which were used to genotype *T. congolense* isolates from livestock in a discrete geographical area of The Gambia. The data showed a high level of diversity with a large number of distinct genotypes, but a deficit in heterozygotes. Further analysis identified cryptic genetic subdivision into four sub-populations. In one of these, parasite genotypic diversity could only be explained by the occurrence of frequent mating in *T. congolense*. These data are completely inconsistent with previous suggestions that the parasite expands asexually in the absence of mating. The discovery of mating in this species of trypanosome has significant consequences for the spread of critical traits, such as drug resistance, as well as for fundamental aspects of the biology and epidemiology of this neglected but economically important pathogen.

## Introduction

African trypanosomes cause disease of high morbidity and mortality in sub-Saharan livestock, and it is estimated that controlling the disease would benefit the agricultural industry by US$1300 million per annum [1]. One of the major causative agents of livestock disease is *Trypanosoma congolense*, which is transmitted by the tsetse fly (*Glossina* spp.). While there is a large body of information on the prevalence, epidemiology and distribution of this parasite, many important basic questions about the biology of this parasite have not been addressed, including the question of whether a system of mating occurs, although this has been shown to occur in the related species *Trypanosoma brucei* [2]. This question is fundamental to our understanding of trypanosome biology and diversity and the evolution of meiosis in these ancient eukaryotes. Drug resistance to the available trypanocides is an increasing problem for *T. congolense* [3], [4] and its spread is a major concern for the sustainable control of the disease. Thus the existence (or not) of mating would also be important at a practical level in terms of the spread of such traits.

The related parasite, *T. brucei*, has a Mendelian system of mating involving meiosis [2], [5]. Mating is a non-obligatory process [6], which occurs in the salivary glands of the tsetse fly vector [7], [8]. *T. brucei*, along with *Trypanosoma cruzi* [9], are the only species of kinetoplastid parasite in which mating has been experimentally studied [5] and, after many years of controversy [10]–[12], there is also strong evidence that some field populations of *T. brucei* undergo frequent mating, while others (the human infective subspecies) show evidence for asexual expansion of particular genotypes [13]. Whether *T. congolense* also undergoes mating is unclear because the current evidence on this question is limited. For example, although monophyletic with *T. brucei*, *T. congolense* is clearly evolutionarily distinct [14], [15], and differs biologically as the life cycle does not involve infection of the salivary glands of the tsetse fly [16]. As mating in *T. brucei* occurs in the salivary glands, there is a reasonable expectation that mating may not occur in *T. congolense*. The available data on *T. congolense* population diversity comes from isoenzyme electrophoresis, and analysis of groups of field isolates has shown genetic heterogeneity with the subdivision of the species into three genetically distinct subgroups, designated as: ‘Savannah’, ‘Kilifi’ and ‘Forest’ [17], [18]. The isoenzyme data have been analysed using a range of criteria to test for the existence of mating [19] and the results indicate high levels of heterozygosity, overrepresentation of identical genotypes and linkage disequilibrium, leading to the conclusion that this species was predominantly clonal. However, the samples used in these analyses originated from diverse regions of Africa and the dates of isolation ranged widely. Thus the observed linkage disequilibrium could be explained on the basis of temporal, genetic or geographic substructuring, and indeed the analysed samples included representatives of the three different subgroups (Savannah, Forest and Kilifi) that are predicted to be genetically isolated. An additional consideration is that all strains used in these studies were expanded by growth in rodents or culture prior to analysis and such expansion is now known to cause potential problems of genotype selection, as illustrated by studies on *T. b. gambiense* [20]. Thus the conclusion that *T. congolense* is predominantly clonal and rarely undergoes genetic exchange is questionable and requires rigorous reassessment using an appropriate sampling strategy, designed to eliminate the potential confounding factors described above.

Clonality, within the context of trypanosome populations, can theoretically arise from two sources [21]. Firstly, there is the potential for true mitotic clonality due to the non-obligatory nature of mating (i.e. genotypes will expand clonally within mammals and in tsetse if there is predominantly transmission without mating). Second, there might be ‘reproductive clonality’, whereby organisms become genetically homogeneous due to being reproductively isolated and mating intragenotypically. However, there is very little evidence for the latter scenario in populations of trypanosomes. Therefore, the description of ‘clonal’ populations in trypanosomes does not mirror that of organisms where sex is an obligatory part of the life cycle such as *P. falciparum* [22], [23], where mating occurs during every transmission through a mosquito vector, and where selfing, and therefore reproductive clonality, readily occurs. In trypanosomes, ‘clonal’ refers to populations with a few predominant genotypes that are largely propagated mitotically, and in which genetic recombination occurs very rarely during vector transmission. Therefore, the aim of this study is to determine the extent of any mating in the tsetse that can be discerned, despite the undoubted mitotic reproduction within mammals and tsetse vectors. By ‘clonal’ we are referring to mitotic clonality i.e. mitotic propagation in the absence of mating in the tsetse.

Analysis of genes from plants, fungi and animals with known roles in meiosis has defined a core set of so called meiotic genes (DMC1, SPO11, MND1, MSH4, MSH5, HOP1, HOP2 & REC8/RAD21 [24]) and orthologues of these have been identified in a number of protozoan parasites including *Giardia intestinalis*, *T. brucei*, *T. cruzi* and *Leishmania major* [25], [26]. The presence of these genes has been used to argue that meiosis occurs in these protists and we have identified orthologues of all these genes in the *T. congolense* genome sequence (http://www.genedb.org/genedb/tcongolense/), showing that the genetic template that may encode meiotic machinery is present, so this species hypothetically has the potential to undergo meiosis and thus have a system of mating.

Based on these considerations, our aim was to determine whether mating takes place in *T. congolense* using a population genetics approach. To do this, a panel of species-specific polymorphic microsatellite markers, were developed and used to genotype *T. congolense* isolates from a large temporally and spatially contiguous set of samples from horses, donkeys and cattle in The Gambia. Analyses of the data obtained allowed the determination of the population structure and the role of mating in the generation of the observed diversity. These findings provide an important new insight into the basic biology of one of the major cattle pathogens of sub-Saharan Africa.

## Results

### Marker analysis

Microsatellite markers were identified by screening of the *T. congolense* genome sequence (http://www.genedb.org/genedb/tcongolense/) with Tandem Repeat Finder software [27], which resulted in the identification of over 4500 loci containing repeats. 25 candidate microsatellite loci were selected based upon repeat fidelity, and these were tested initially against a reference panel of *T. congolense* isolates to determine the level of polymorphism and potential subgroup-specificity, and against *T. brucei* and *T. vivax* genomic DNA to ensure species-specificity. Seven microsatellites (TCM1-7) showed no amplification with *T. brucei* or *T. vivax* DNA, but polymorphic amplified products were detected in all of the *T. congolense* Savannah subgroup used as a reference panel (14 isolates from seven countries). However, there was no product detectable by gel electrophoresis for TCM3 for any of the three *T. congolense* Forest subgroup isolates, suggesting that this locus either has a polymorphism in the primer site, or is not present in the *T. congolense* Forest genome. In addition, no product was detectable for TCM1, TCM2, TCM3 or TCM4 for the one *T. congolense* Kilifi subgroup sample tested, also suggesting polymorphism or absence of the loci. This indicates that TCM3 may be suitable for use as a marker to differentiate between *T. congolense* Savannah, and *T. congolense* Forest and Kilifi.

A total of 535 field samples from the Central region of the Gambia (276 horses, 75 donkeys, 184 cattle) were tested for the presence of *T. congolense* using PCR of the species-specific minichromosomal satellite repeat marker and 133 samples were PCR positive for *T. congolense* Savannah (80 horses, 26 donkeys and 27 cattle – a prevalence of 28.9% in horses, 34.6% in donkeys and 14.6% in cattle). No *T. congolense* Forest infections were detected (from hereon ‘*T. congolense*’ refers to *T. congolense* Savannah). Eighty four of the 133 *T. congolense* positive samples amplified for all seven microsatellite loci (51 horses, 12 donkeys and 21 cattle). Polymorphism was evident for all seven loci and ranged from six alleles for TCM4 to 13 alleles for TCM5 (Table 1; for allele sizes and allele frequency data see Tables S3 and S4, respectively). There were 17 samples with detectable mixed infections (as defined by Peak Scanner detecting more than two alleles at at least one locus) giving a prevalence of mixed infections within the *T. congolense* positive samples of 20.2%, with only one sample being identified as mixed at more than one locus. While this is the most likely explanation of samples with three alleles at a locus, the possibility of triploidy cannot be ruled out, as this has been observed in *T. brucei* genetic hybrids for some markers after mating events [28], [29]. Treating the samples as a single population, all seven loci exhibited heterozygote deficiency based on a lower value of observed heterozygosity compared to expected (Table 1) resulting in a positive inbreeding coefficient ranging from 0.29 to 0.77. These results are unlikely to be due to allele drop out as the method used for DNA and marker amplification had previously been shown to eliminate this problem [30].

**Table 1:** Marker polymorphism, heterozygosity and selfing in the Gambian population (n = 89*).

Locus	Na†	Ho	He	F_IS_	s	p	Ne	No
**TCM1**	8	0.25	0.73	0.66	0.79	0.28	6.9	1
**TCM2**	9	0.17	0.71	0.77	0.87	0.32	9.2	1
**TCM3**	9	0.51	0.77	0.33	0.49	0.14	1.8	1
**TCM4**	6	0.25	0.56	0.58	0.73	0.21	3.9	0
**TCM5**	13	0.42	0.83	0.50	0.67	0.23	4.6	0
**TCM6**	9	0.51	0.71	0.29	0.45	0.12	1.3	0
**TCM7**	8	0.49	0.75	0.34	0.51	0.15	1.9	4

* see materials and methods.

† Abbreviations: Na = number of alleles, Ho = observed heterozygosity, He = expected heterozygosity, F_IS_ = inbreeding coefficient, s = predicted selfing rate, p = frequency of predicted null alleles, Ne = number of predicted homozygous nulls, No = number of observed homozygous nulls.

### Genetic analysis

**Figure 1:**
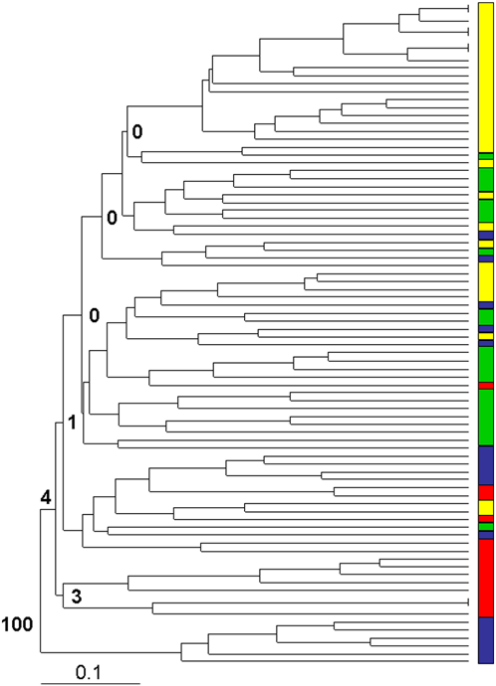
Dendrogram of *T. congolense* multilocus genotypes (MLGs)(n = 84). Bootstrap values were calculated from 100 iterations and are shown at relevant nodes. The coloured bars indicate which subpopulation the individual is assigned to by STRUCTURE (red = W, green = X, blue = Y, yellow = Z).

To further examine the level of diversity in the population, multilocus genotypes (MLGs) were derived for each isolate from the genotyping with the seven microsatellite markers and these were used to construct a dendrogram of similarity, by calculating Jaccard's coefficient for all pairwise comparisons (Fig. 1). The Gambian *T. congolense* population formed one large group, with little bootstrap support for differentiation within the sample set, except for one small subgroup (samples 111, 1065, 2021, 3005, 3022 and 3033) with strong bootstrap support (100, Fig 1). The values for all other key nodes within the cluster were less than four. A total of 80 distinct MLGs were identified in the 84 samples indicating very little evidence for clonal expansion of particular genotypes. The four duplicated genotypes were tested for clonality using MLGsim [31], and the P_sex_ values for each MLG expansion were not significant at the p<0.01 threshold for three of the duplicated genotypes (samples 119 & 2081, 3008 & 3011, 3010 & 3021), which indicated that the repeated MLGs had arisen through independent recombination (data not shown). The fourth duplicate (samples 103 and 2010) was highly significant at the p<0.01 threshold and fell well outside the distribution of simulated values, suggesting that it had arisen through clonal replication. The dendrogram shows a high level of genetic diversity between isolates, albeit with many common alleles, that is difficult to account for solely by mutation and asexual reproduction.

Allele frequencies and MLGs were then used to analyse linkage disequilibrium, agreement with Hardy-Weinberg predictions and the extent of any population differentiation or substructuring. When the population was analysed for agreement with Hardy-Weinberg predictions, there was significant deviation from Hardy-Weinberg equilibrium at all loci (Table S5). When linkage equilibrium was analysed between pairwise combinations of loci (Table S6), more than half (13/21) loci combinations were in linkage disequilibrium. To estimate the level of linkage disequilibrium in the population as a whole, the Standardised Index of Association (I^S^_A_; an average measure over all loci) was estimated and the I^S^_A_ value indicates that the population is in linkage disequilibrium (Table 2; I^S^_A_ = 0.039, V_D_>L), although this value is close to the zero value for linkage equilibrium (see Materials and Methods for a detailed explanation of V_D_ and L). To test if this departure from LE was due to the presence of MLG duplicates, these were removed, but the population remains in linkage disequilibrium (data not shown), thus showing that it does not have an epidemic structure (where underlying mating is masked by the short-term clonal expansion of a predominant genotype [10]).

**Table 2:** Standardised Index of Association for the Gambian *T. congolense* population as a whole (All samples), and when separated into subpopulations by STRUCTURE (W, X, Y, Z).

Population	n*	I^S^_A_	P value	V_D_	L	LE/LD
All samples	84	0.039	0.01	2.13	1.70	LD
W	14	0.095	0.01	1.52	1.19	LD
X	27	0.006	**0.45**	1.03	1.16	**LE**
Y	17	0.074	0.01	1.54	1.34	LD
Z	26	0.067	0.01	1.94	1.63	LD

* Abbreviations: n = number of samples, I^S^_A_ = standardised index of association, V_D_ = variance of pairwise differences, L = 95% critical value for V_D_, LE = linkage equilibrium (shown in bold type), LD = linkage disequilibrium.

**Figure 2:**
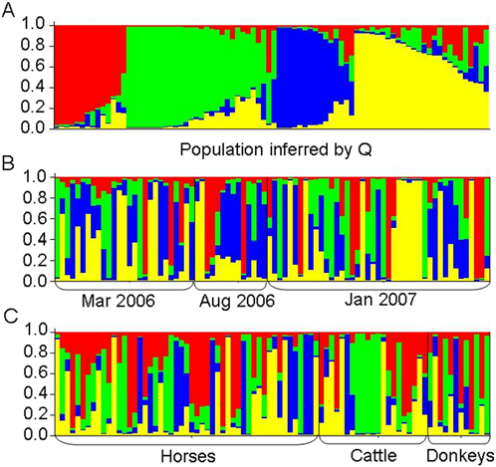
Estimated population substructure (n = 84). Each individual is represented by a vertical bar, divided into four coloured segments that represent the proportion of the genotype for that individual that derives from each of the four clusters (Q), four being the estimated number of subpopulations as detected by STRUCTURE. A is ordered by Q (red = W, green = X, blue = Y, yellow = Z). In B and C, individuals are ordered along the x axis according to sampling date (B) or host species (C).

The lack of linkage equilibrium and deviation from Hardy-Weinberg could arise for a number of reasons other than limited genetic exchange and these include cryptic speciation, temporal, host or micro-geographic substructuring as well as genetic substructuring as a result of immigration. To investigate potential substructuring, STRUCTURE software was employed [32]. Permutations of the number of clusters (K) from 1–10 (a cluster being equivalent to a subpopulation) were evaluated for the *T. congolense* data set using the method of Evanno et al [33] (Fig. S1). Based on this analysis there is evidence for substructuring and the most likely number of subpopulations is four (W, X, Y, Z; Fig. 2). The model used in the simulations assumed correlated allele frequencies within populations, and admixture allowed for mixed ancestry within individuals. Each vertical bar in Fig. 2 represents an individual and the amount of each of the four colours in an individual indicates the proportion of that individual's genetic makeup that derives from each of the four subpopulations as assigned by STRUCTURE. Therefore, the subpopulations identified are suggested by STRUCTURE to be distinct, but there has been a degree of genetic exchange between them, as evidenced by the individuals with mixed ancestry. If subpopulations are pre-defined based on host or date of sampling they do not correspond to the subpopulations inferred by STRUCTURE (Fig. 2, compare A with B and C) and so these parameters can be eliminated as the basis for the substructuring. Other data [34] available (place of sampling, packed cell volume of host, diagnosis by microscopy, and age of host) also did not correlate with the four subpopulations. Each of the inferred subpopulations was then tested for agreement with Hardy-Weinberg predictions and although none of the four subpopulations show agreement for all loci, some loci are in agreement with the values predicted for a randomly mating population (Table S5). Analysis of linkage equilibrium (LE) between alleles at all pairwise combinations of loci showed that only 8/21 combinations are in linkage equilibrium when all isolates are treated as a single population, but in the sub populations the number in LE doubled (W, 16/21; X, 18/21; Y, 15/21; Z, 16/21; Table S6). To further test the level of linkage disequilibrium in the subpopulations, the index of association was measured (I^S^_A_; Table 2) and the largest subpopulation (X) shown to be in LE, thus indicating frequent mating within this population. As the programme tests the observed data against 10,000 simulations using the same sample size, the possibility of a type II error is very low, although cannot be totally excluded.

The pairwise F_ST_ values [35] between the inferred subpopulations (Table 3) all indicate moderate genetic differentiation (values ranging from 0.078 to 0.146), aside from the comparison between Y and Z subpopulations, which shows a greater genetic differentiation (F_ST_ = 0.178). This is in contrast to the F_ST_ values if the population is partitioned into sub-populations by sampling criteria, such as host species or date of sampling (Table 3), where the values are all very low (<0.024) and indicate little genetic differentiation. To further test this genetic differentiation, Nei's genetic distance was measured between the subpopulations and the values are dramatically greater (0.368–0.844) compared to those partitioned by sampling criteria (0.05–0.085; Table 3). With the available data the basis behind the cryptic subpopulations identified by STRUCTURE is as yet unknown.

**Table 3:** Pairwise values of Wright's fixation index (F_ST_; above diagonal) and Nei's genetic distance (*D*; below diagonal) between subpopulations of *T. congolense* as defined by STRUCTURE (W, X, Y, Z), or by host species (Horse, Donkey, Cow) and by sample date (Mar 2006, Aug 2006, Jan 2007).

	W	X	Y	Z
W	–	0.078	0.100	0.146
X	0.409	–	0.094	0.109
Y	0.582	0.500	–	0.178
Z	0.615	0.368	0.844	–

	Horse	Donkey	Cow
Horse	–	0.009	0.015
Donkey	0.050	–	0.013
Cow	0.075	0.058	–

	Mar 2006	Aug 2006	Jan 2007
Mar 2006	–	0.018	0.009
Aug 2006	0.085	–	0.024
Jan 2007	0.050	0.118	–

The data show that the *T. congolense* population in The Gambia is genotypically diverse with few identical genotypes. The data is inconsistent with clonality, but there are barriers to complete random mating. There is very little genetic differentiation between the populations in different hosts or those separated temporally, as measured by F_ST_, clustering analysis or Nei's genetic distance. Analysis of the genotype and allele frequency data shows that the population deviates from Hardy-Weinberg expectations and is in linkage disequilibrium as measured by the index of association. These departures from the null hypothesis of panmixia cannot be explained by either temporal or host substructuring but analysis by the programme STRUCTURE supports the existence of four ‘cryptic’subpopulations, which by measurement of pairwise F_ST_ are moderately differentiated. One of these subpopulations is in linkage equilibrium thus supporting the occurrence of frequent mating.

## Discussion

The sample set presented in this study provided the opportunity to examine the issue of whether genetic exchange is occurring in *T. congolense* using a geographically discrete population, in contrast to previous studies [11], [19] where samples from a wide geographic area were analysed. The use of blood samples on FTA filters, combined with Whole Genome Amplification (WGA) and PCR technology [30], allowed the direct analysis of a trypanosome population that is not separated by time (at least, all samples were collected within one calendar year) or geography, and also avoided the potential for selection of genotypes by growth of the parasites prior to analysis. The population does not have any of the key features of clonality, such as fixed heterozygosity and overrepresentation of identical genotypes [11], [36], which have been observed in previous studies using isoenzyme markers [11]. Only four MLG duplicates were identified, three of which are suggested to have arisen independently via recombination according to P_sex_ values, which, at the very least, indicates that the population structure is not epidemic as has been shown for *T. brucei* populations [13]. However, barriers to unrestricted genetic exchange were detected as there is a significant heterozygote deficit at all loci, and therefore the population is not in Hardy-Weinberg equilibrium. There is also linkage disequilibrium as measured between all pairwise loci or at a global level for the population using I^S^_A_. Collectively, this evidence suggests that the population is not panmictic. In contrast, the lack of distinct subgroups from the similarity analysis coupled with the high proportion of unique MLGs suggests that genetic exchange may be occurring frequently.

A number of explanations for the heterozygote deficit and low level of linkage disequlibrium can be considered. The first, and most likely, is the Wahlund effect, whereby we are actually analysing several distinct populations as one, and we provide evidence for this. STRUCTURE software detected ‘cryptic’ substructuring within the *T. congolense* population (cryptic in this sense referring to the subpopulations not correlating to any measured parameter set, such as host or time). The results indicated that the most likely number of cryptic subpopulations was four, and these differed significantly by F_ST_ and Nei's genetic distance, previously masked due to the subpopulations being mixed when arbitrarily partitioned into populations by sampling date or host species. One of these subpopulations (X) shows linkage equilibrium and therefore evidence for frequent mating. It should be stressed that while linkage disequilibrium is detected in the population as a whole and in some subpopulations, the values of the index of association are all close to zero (I^S^_A_ = 0.074–0.095), indicating a limited level of disequilibrium.

An alternative, but improbable, explanation for the linkage disequilibrium is that this population is clonal. However, aside from linkage disequilibrium, none of the features of the population are consistent with clonality. Firstly, as described above, common features of clonal trypanosomatid populations are the observation of a large proportion of identical or very similar genotypes and secondly a high value for the Index of Association. For example, in the related human infective *T. b. gambiense*, which is considered clonal [37], microsatellite genotyping of a population from the Democratic Republic of Congo identified only 17 distinct MLG's from a sample of 37 isolates and the index of association was nearly five times higher (I^S^_A_ = 0.35–0.44 based on a re-analysis of data in [21]) than that observed here for the *T. congolense* population. An extreme possibility that could potentially explain the genetic diversity combined with linkage disequilibrium is a clonal population with a high rate of mutation leading to many diverse clones due to the high mutation rate. This scenario has not been observed in clonal populations of trypanosome species previously (for example [21], [38]), and furthermore, one would predict an excess of heterozygotes based on this hypothesis in contrast to the deficit observed in *T. congolense*. Clonality and high diversity in a temporally and spatially contiguous data set has not been demonstrated in trypanosomes; one study that did provide evidence for considerable diversity in clonal populations of *T. cruzi* [39] is not comparable due to the use of a sample set (n = 25) covering five calendar years and 10 geographic localities. Therefore, the data on *T. congolense* presented here are not consistent with a high mutation rate alone leading to the level of genetic diversity observed in our data set.

The observed heterozygote deficit at all loci could be explained by allele dropout, null alleles, selfing, or a level of non-obligatory mating. It is possible that the WGA and low level of parasitaemia lead to a failure of one allele to amplify by PCR, and therefore an excess detection rate of pseudo-homozygotes. However, we have shown that this does not occur if three pooled WGA reactions are used, and so this explanation is unlikely [30]. In a range of organisms, null alleles have been identified and with our data these could arise as a result of polymorphism in the primer sites leading to a lack of marker amplification. We estimated the frequency of null alleles that would result in the restoration of Hardy Weinberg equilibrium and, based on this frequency, we calculated the number of samples that would be homozygous for the null allele at each locus (Table 1) and so not yield an amplicon. For markers TCM3, 6 and 7, the numbers of predicted homozygous nulls is low and with our sample size might not be detected. However, the number predicted for markers TCM 1, 2, 4 and 5 are all >3.88 and so should be detected. The number of observed homozygous nulls detected for each of these loci (1 or 0) is well below that predicted. Therefore, null alleles are an improbable explanation for the heterozygote deficit in our data. Selfing or mating between closely related individuals would result in a heterozygote deficit and while, for example, selfing occurs at a significant level in *Plasmodium falciparum* [23], the sexual stage of the life cycle in that parasite is obligatory. Both outcrossing and selfing has been described in *T. brucei* in laboratory crosses [40], [41], but the products of selfing have only been identified when cross fertilisation occurs, and not observed when a single strain is used to infect tsetse. This has not been investigated in field populations, but predicted selfing rates were calculated for our data set (Table 1) and shown to need to be high to account for the observed heterozygote deficit, and furthermore, selfing would have to occur in the absence of inter-genotype mating. This is not consistent with laboratory data for *T. brucei*, but the possibility of selfing having an influence upon the population genetics of *T. congolense* cannot be ruled out. An alternative possibility is that non-obligatory mating, as described for *T. brucei* laboratory crosses [6], occurs and so some strains will undergo recombination but others will be transmitted without any change in genotype. While this could provide an explanation for the heterozygote deficit, a significant frequency of identical genotypes would be predicted but this is not observed. Based on these considerations selfing seems the most likely explanation but deserves further investigation.

Aside from the conclusion that the diversity observed can only be accounted for by mating taking place, a striking finding is the evidence for substructuring, although the basis for this is not evident from our data set. One explanation is that there has been recent migration leading to subpopulations that have not fully admixed. The subpopulations could also reflect biological differences of the parasite, for example vector species preference. It is known that there are at least two species of *Glossina* present in the study region of The Gambia [42], although further work would be necessary to investigate these possibilities. Alternatively, there could be small scale geographical substructuring with very local cycles of transmission. We cannot address this with the current sample set as fine details of geographical origin were not recorded for the infected animals that were presented at the clinic.

Our analysis of the genotype data cannot be reconciled with an asexual and clonal population structure but strongly supports the occurrence of mating as the mechanism for generating the high levels of observed diversity. The departures from the predicted genotype frequencies and the heterozygote deficit in the whole population suggest that mating is not occurring totally at random, but the population is genetically sub divided and mating is occurring at high frequency in one of these sub-populations. The basis for this sub-division requires further investigation but could arise from recent immigration or from micro-geographic sub-structuring based on highly focal vector-host cycles. The discovery of mating for this highly prevalent and pathogenic parasite of livestock has complex but considerable consequences for the spread of traits such as drug resistance. Our finding also has implications for the evolution of sexual recombination in eukaryotes; some African trypanosome species are evidently clonal and either never underwent, or have dispensed with, a sexual cycle (*T. b. gambiense* and *T. vivax*), whereas others clearly undergo frequent mating (*T. congolense* and *T. b. brucei*). Therefore, trypanosomes provide unique potential for examining the origin and evolution of meiosis in ancient eukaryotes.

## Materials and Methods

Samples in this study were a byproduct of veterinary screening of animals (horses and donkeys) voluntarily presented at the Gambian Horse and Donkey Trust (GHDT) clinic or mobile survey unit, or as part of routine sampling of the cattle herd at the International Trypanotolerance Centre (ITC) field station. All samples were collected by trained veterinarians or paraveterinarians, and animals diagnosed or suspected to be infected with trypanosomes were treated with trypanocides. For the purposes of this study, a spare 200 µl fraction of the blood collected by venupuncture into a heparin vacutainer was spotted onto an FTA® filter (Whatman) and allowed to air-dry. This sampling approach avoided any potential selection of the parasites by growth in rodents prior to analysis. The samples were collected in March and August 2006, and January 2007 from locations within a 50 km radius in the Central River District of The Gambia. Equine samples (n = 351; 276 horses and 75 donkeys) were taken at the GHDT clinic at Sambelkunda in Niamina East District (n = 254) or at local villages (n = 73) and ITC field station at Bansang (n = 24). Cattle samples were taken from the N'dama herds of the ITC at its field sites near Touba and Sambelkunda in Niamina East District (n = 169) and from a nearby village (n = 15).

All field samples were initially tested by PCR with primers directed against minichromosomal satellite repeats which are specific to each species or subgroup (*T. congolense* Savannah, *T. congolense* Forest, *T. vivax* and *T. brucei* [43]). Whole Genome Amplification (WGA) reactions were carried out on all *T. congolense* positive samples as described previously [30], with two punches from each FTA disc used as substrate in each reaction, and pooling of three independent reactions to minimise allele dropout (a failure of one allele to amplify by PCR).

Microsatellite markers were identified by screening the available genome sequence (http://www.genedb.org/genedb/tcongolense/) using Tandem Repeat Finder software [27]. Over 4500 loci were identified that contained repeats and these were ranked based on their repeat fidelity within each locus, in order to identify a subset of loci with conserved repeat motifs. Nested oligonucleotide primers were designed to the unique flanking sequence of 25 loci with high fidelity repeat motifs, and these were tested against a *T. congolense* reference panel for species (or potential subgroup) specificity and allelic polymorphism. The reference panel consisted of DNA from 18 *T. congolense* isolates, as well as DNA from *T. brucei* TREU 927 and *T. vivax* ILRAD V-34. Several of these reference samples were kindly provided by Professor Wendy Gibson, Bristol University (GAM 2, WG 81, TSW 13, TSW 103, ANR 3, CAM 22 and WG 5). The panel consisted of 14 *T. congolense* Savannah, three *T. congolense* Forest, and one *T. congolense* Kilifi (Table S1). The screening of this panel by PCR amplification resulted in the identification of seven polymorphic microsatellite markers (TCM 1–7; for primer sequences see Table S2), which were used in the analysis of the field population. The scaffolds encoding the microsatellite markers were ordered and orientated against the individual chromosomes of the *T. brucei* reference genome, using postulated conserved gene order in the absence of further karyotype information. Six of the seven markers mapped to a different *T. brucei* chromosome whilst the seventh marker is located in a subtelomeric region. We are therefore confident that the loci are not physically linked. Conditions for all primer sets for both rounds of PCR were 28 cycles of 50 seconds at 95°C, 50 seconds at 52°C and 1 minute at 65°C. As the parasitaemias of the infected samples were low, and despite whole genome amplification, it was necessary to undertake nested PCR for microsatellite amplification. For the second round PCR, a 1/100 dilution of the first round product was used as template.

For the second round nested PCRs, one primer included a 5′ FAM or HEX modification, allowing separation of labelled PCR products by size (Dundee Sequencing Service; http://www.dnaseq.co.uk), and determination of allele size to an accuracy of one bp using Peak Scanner v1.0 software (Applied Biosystems). Each allele was designated a unique number for each locus, and a multilocus genotype (MLG) defined by the specific combination of alleles at all seven loci. If a sample contained more than two alleles, it was defined as a mixed infection and the predominant allele or alleles for each marker were chosen for identifying the MLG, with predominance being based on peak area from the Peak Scanner output as described previously in other systems [44], [45].

The probability of null alleles playing a part in the observed heterozygote deficit was analysed by calculating the expected number of homozygous nulls based on the heterozygosity data for the seven markers. The samples included in this analysis were those that amplified for 4 or more loci (n = 89; see Table S2). The majority of samples that were positive for *T. congolense* by minichromosomal satellite repeat PCR (n = 133) amplified either for all 7 microsatellite markers (n = 84), or for none (n = 25)(Fig. S2). It was not possible to differentiate the non-amplification in those samples that amplified at no or fewer than 3 loci (n = 44) between non-amplification due to null alleles or that due to sensitivity threshold because of low number of parasites. Furthermore, if one assumes there is an even distribution of null alleles among the seven markers, which is reasonable given that the consistently high F_IS_ values observed across the seven unlinked markers, then one would not expect the binomial distribution of samples graphed against number of markers giving amplification (Fig. S2). Therefore, for the analysis of null alleles (Table 1), 89 samples were used that amplified for 4 or more loci; for all other analysis only the 84 samples that amplified for all seven markers were used. The analysis of null alleles and predicted selfing rate was determined using the following calculations: s = (2F_IS_)/(1+F_IS_)[46], frequency of predicted null alleles p = (He−Ho)/(1+He)[47], and number of predicted homozygous nulls Ne = p^2^n, where n = number of samples.

Analysis of MLGs used Clustering Calculator (http://www2.biology.ualberta.ca/jbrzusto/cluster.php), which generated a Phylip Drawtree string (unweighted arithmetic average clustering method, and Jaccard's similarity coefficient), which was converted into a dendrogram by Treeview (http://taxonomy.zoology.gla.ac.uk/rod/treeview.html)[48]. Clustering Calculator generated the bootstrap values for dendrograms, using 100 iterations. Jaccards coefficient was used to allow comparison with other published parasite population studies where it has been used extensively [44], [45], [49]. Data on marker polymorphism and heterozygosity (observed and expected), inbreeding coefficient (F_IS_), selfing rate and Wright's fixation index (F_ST_) were calculated using GenAlex [50]. Hardy-Weinberg equilibrium and linkage disequilibrium between paired loci were examined using Genepop 3.2 [51]. The Standardised Index of Association (I^S^_A_) was used to measure associations between alleles at all pairwise combinations of loci and was calculated using LIAN 3.5 [52]. The genotypes were treated as described by Stevens and Tibayrenc [53]. This statistical test determines the variance (V) of the distribution of the number of shared alleles between all pairwise comparisons of the MLGs and compares this to the variance in the distribution of the number of shared alleles from 10,000 simulations where the alleles at each locus are randomly shuffled (the null hypothesis of random mating). The index is expressed as one minus the ratio of the two values of the variance and so is zero or negative when the population is undergoing random mating but is positive if there is allele association or linkage disequilibrium. The conclusion can be statistical tested by measuring the 95% confidence limit (L) between the observed and expected values such that if V>L the null hypothesis can be rejected and from simulations a probability (p) of this measured. The advantage of using this measure for assessing linkage equilibrium is that it combines measures over all loci as a single estimate and it allows a robust statistical test for the agreement with the null hypothesis of panmixia. The likelihood of duplicated genotypes occurring due to sexual recombination was examined using the MLGsim programme [31], which is a further development of that described by Tibayrenc et al [11], whereby P_sex_ values are calculated for each replicated MLG and compared with values generated by 10,000,000 simulations for a population of the same size. Population substructuring was investigated using STRUCTURE [32], which utilises a Bayesian approach to infer the number of subpopulations or clusters (K) within a data set. The model used assumed that allele frequencies were correlated within populations, and admixture allowed for mixed ancestry within individuals. Ten independent simulations were carried out at each value of K (K = 1–10), with a burn in period of 10000 iterations and 10000 replications. The most likely number of estimated subpopulations was determined on the basis of the maximum value of the ad hoc parameter ΔK [33] for values of K from one to ten, using the second order rate of change of the likelihood function between successive values of K (Ln P(D)) (SI Figure 1). STRUCTURE outputs were also generated by pre-allocating samples to subpopulations as defined by host species and time collected, in order to look for correlation between these parameters and the subpopulation allocation by STRUCTURE.

## Supporting Information

Figure S1 The most parsimonious estimate of the number of subpopulations (K) was determined to be four on the basis of the maximum value of the ad hoc parameter delta K, which was calculated for values of K from one to ten, using the second order rate of change of the likelihood function between successive values of K (K = (Ln P(D)) in the STRUCTURE output - for details on the calculation of delta K see [30]).
(0.03 MB DOC)

Figure S2 Number of Gambian isolates positive by diagnostic PCR that amplified for microsatellite markers, graphed by number of loci amplifying per sample.
(0.07 MB DOC)

Table S1 Date and place of origin for reference panel of *T. congolense* isolates.
(0.04 MB DOC)

Table S2 Oligonucleotide primer sequences (5′-3′) for microsatellite markers.
(0.04 MB DOC)

Table S3 Allele sizes detected at 7 microsatellite markers for the Gambian *T. congolense* population.
(0.47 MB DOC)

Table S4 Allele frequencies at 7 microsatellite markers for the Gambian *T. congolense* population. The upper, non-italicised data are the frequencies for the 84 samples that amplified for all 7 loci. Samples in italics are frequencies for all samples (n = 133), including those that did not amplify for all loci.
(0.07 MB DOC)

Table S5 Hardy-Weinberg analysis for *T. congolense* populations including all samples that amplified for 7 microsatellite markers, and samples from subpopulations as defined by STRUCTURE. * P-value; those not significant at the 0.05 level are shown in bold.
(0.04 MB DOC)

Table S6 Linkage disequilibrium (bold type) between pairwise loci in *T. congolense* samples from The Gambia, analysed for all samples that amplified for 7 microsatellite markers and samples from subpopulations as defined by the STRUCTURE programme.
(0.07 MB DOC)
