# Supplementary figures and images for: Discovery of Mating in the Major African Livestock Pathogen Trypanosoma congolense
Source: PLoS One. 2009 May 15;4(5):e5564. doi: 10.1371/journal.pone.0005564 (PMC2679202; doi:10.1371/journal.pone.0005564)

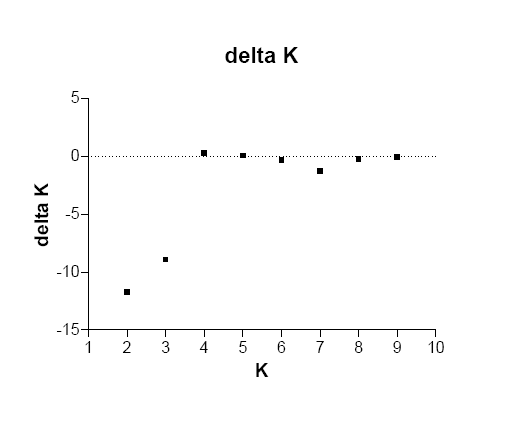

Supplement: Figure S1 — The most parsimonious estimate of the number of subpopulations (K) was determined to be four on the basis of the maximum value of the ad hoc parameter delta K, which was calculated for values of K from one to ten, using the second order rate of change of the likelihood function between successive values of K (K = (Ln P(D)) in the STRUCTURE output - for details on the calculation of delta K see [30]). (0.03 MB DOC) [file pone.0005564.s001.doc]
