# Supplementary material for: Discovery of Mating in the Major African Livestock Pathogen Trypanosoma congolense
Source: PLoS One. 2009 May 15;4(5):e5564. doi: 10.1371/journal.pone.0005564 (PMC2679202; doi:10.1371/journal.pone.0005564)
Supplement: Table S1 — Date and place of origin for reference panel of T. congolense isolates. (0.04 MB DOC) [file pone.0005564.s003.doc]

| Isolate | Date | Origin | Type |
| --- | --- | --- | --- |
| GAM 2 | 1977 | The Gambia, cow | Savannah |
| WG 81 | 1981 | Kenya, goat | Savannah |
| TSW 13 | 1975 | Liberia, pig | Savannah |
| TREU 1457 | 1967 | Nigeria, cow | Savannah |
| TREU 1676 | 1977 | Tanzania, gazelle | Savannah |
| TREU 1627 | 1977 | Gambia, cow | Savannah |
| TREU 1894 | 1981 | Zambia, dog | Savannah |
| TREU 1885 | 1981 | Zambia, dog | Savannah |
| TREU 2037 | 1981 | Zambia, dog | Savannah |
| ILRAD 2921 | 1953 | Burkina Faso, cow | Savannah |
| ILRAD 2829 | 1971 | Burkina Faso, cow | Savannah |
| KETRI 2885 | 1971 | Tanzania, lion | Savannah |
| KETRI 2883 | 1985 | Kenya, cow | Savannah |
| KETRI 2880 | 1983 | Burkina Faso, cow | Savannah |
| TSW 103 | 1977 | Liberia, pig | Forest |
| ANR 3 | 1988 | The Gambia, tsetse | Forest |
| CAM 22 | 1984 | Cameroon, goat | Forest |
| WG 5 | 1980 | Kenya, goat | Kilifi |
