# Supplementary material for: Discovery of Mating in the Major African Livestock Pathogen Trypanosoma congolense
Source: PLoS One. 2009 May 15;4(5):e5564. doi: 10.1371/journal.pone.0005564 (PMC2679202; doi:10.1371/journal.pone.0005564)
Supplement: Table S2 — Oligonucleotide primer sequences (5′-3′) for microsatellite markers. (0.04 MB DOC) [file pone.0005564.s004.doc]

| First round primer pairs | |
| --- | --- |
| TCM1A | TACAAATGACTGTAGAGCGGC |
| TCM1B | CTGTGTGTATAATGATTCATTCG |
| TCM2A | GGTAAGACAAAGTTGTGGGTG |
| TCM2B | ATGTGACCGATGCTCCGAAC |
| TCM3A | TCTATTGTTCACGTCTCGTG |
| TCM3B | ACTCATTGCATAAAGGCTAG |
| TCM4A | CTTAACGCTGCTTCAGTAGC |
| TCM4B | AGTACACACGACTTCACCTCC |
| TCM5A | CAATGGTTCAATAAGCGCACC |
| TCM5B | AAGGCAAGTAAGTTACGC |
| TCM6A | GAATGCGAGACCTGCTTCTTGG |
| TCM6B | CATTTAGACTCTCACTTTCCG |
| TCM7A | GTGTAGTTTGTTATACTTCG |
| TCM7B | GTTAAATACTTGTGAGAGCCAGC |
| Second round primer pairs | |
| TCM1C | CTAGAAGCGAGTAACAGCC |
| TCM1D | AAGGGTTCGTACCACAGCCC |
| TCM2C | CAGTCATGTATATGTTTGTG |
| TCM2D | CCTGAAATGGGTCTACTGAG |
| TCM3C | CATGCTCTTAGGTTCCATCGG |
| TCM3D | AGCATCCGACATTGAAACGAC |
| TCM4C | GTCTCTTTCCGCACAGTGAC |
| TCM4D | GGGGGAAGATATTAAAGACAC |
| TCM5C | CTTCCACGAGTCCCTAATCGAC |
| TCM5D | TTGCTCACTGTCAAGGCGTGC |
| TCM6C | AACCACCACTTCCGTGCACCGG |
| TCM6D | CCATGAGCTTTATGCGACCTCTAC |
| TCM7C | TCATAGAGGCAAGTGCGTAGC |
| TCM7D | CCAGAATAAGAATACTTACTGC |
