# Supplementary material for: Discovery of Mating in the Major African Livestock Pathogen Trypanosoma congolense
Source: PLoS One. 2009 May 15;4(5):e5564. doi: 10.1371/journal.pone.0005564 (PMC2679202; doi:10.1371/journal.pone.0005564)
Supplement: Table S3 — Allele sizes detected at 7 microsatellite markers for the Gambian T. congolense population. (0.47 MB DOC) [file pone.0005564.s005.doc]

| Sample | date^*^ | Sp^†^ | TCM1^‡^ | | TCM2 | | TCM3 | | TCM4 | | TCM5 | | TCM6 | | TCM7 | |
| --- | --- | --- | --- | --- | --- | --- | --- | --- | --- | --- | --- | --- | --- | --- | --- | --- |
| 6 | Mar06 | H | 173 | 180 | 191 | 191 | 171 | 171 | 153 | 153 | 157 | 157 | 175 | 188 | 176 | 179 |
| 14 | Mar06 | H | 176 | 180 | 177 | 177 | 171 | 185 | 153 | 156 | 157 | 165 | 180 | 185 | 179 | 185 |
| 16 | Mar06 | H | 176 | 180 | 181 | 181 | 171 | 182 | 153 | 153 | 150 | 150 | 180 | 185 | 179 | 182 |
| 19 | Mar06 | H | 180 | 180 | 177 | 181 | 175 | 191 | 153 | 167 | 153 | 153 | 169 | 180 | 179 | 182 |
| 29 | Mar06 | H | 176 | 176 | 181 | 181 | 171 | 185 | 156 | 160 | 165 | 165 | 180 | 185 | 176 | 179 |
| 34 | Mar06 | H | 180 | 180 | 181 | 181 | 171 | 171 | 156 | 156 | 148 | 148 | 180 | 180 | 185 | 185 |
| 35 | Mar06 | H | 178 | 178 | 177 | 177 | 171 | 171 | 156 | 156 | 157 | 167 | 169 | 185 | 176 | 179 |
| 36 | Mar06 | H | 200 | 200 | 181 | 181 | 171 | 171 | 153 | 160 | 153 | 153 | 185 | 185 | 176 | 176 |
| 40 | Mar06 | D | 176 | 176 | 181 | 181 | 171 | 171 | 160 | 160 | 153 | 161 | 180 | 185 | 176 | 176 |
| 43 | Mar06 | H | 180 | 180 | 181 | 181 | 175 | 175 | 156 | 163 | 142 | 161 | 175 | 175 | 176 | 176 |
| 48 | Mar06 | D | 170 | 170 | 185 | 185 | 185 | 191 | 153 | 153 | 157 | 157 | 185 | 185 | 176 | 176 |
| 59 | Mar06 | D | 180 | 180 | 177 | 181 | 175 | 191 | 153 | 153 | 148 | 148 | 169 | 180 | 176 | 182 |
| 62 | Mar06 | D | 176 | 210 | 177 | 177 | 148 | 171 | 156 | 156 | 153 | 161 | 185 | 185 | 179 | 179 |
| 80 | Mar06 | H | 176 | 176 | 177 | 177 | 171 | 171 | 156 | 156 | 148 | 148 | 185 | 185 | 179 | 179 |
| 93 | Mar06 | D | 176 | 180 | 177 | 181 | 171 | 171 | 156 | 156 | 175 | 175 | 180 | 188 | 176 | 176 |
| 100 | Mar06 | H | 176 | 176 | 177 | 177 | 171 | 191 | 156 | 170 | 153 | 161 | 195 | 195 | 176 | 179 |
| 103 | Mar06 | H | 180 | 180 | 181 | 191 | 200 | 200 | 156 | 156 | 150 | 150 | 173 | 175 | 176 | 182 |
| 111 | Mar06 | D | 170 | 180 | 179 | 183 | 175 | 175 | 156 | 160 | 161 | 161 | 180 | 180 | 173 | 173 |
| 118 | Mar06 | H | 178 | 178 | 177 | 177 | 171 | 185 | 156 | 156 | 157 | 157 | 180 | 185 | 179 | 185 |
| 119 | Mar06 | H | 176 | 176 | 177 | 177 | 171 | 171 | 156 | 156 | 157 | 175 | 180 | 185 | 179 | 179 |
| 127 | Mar06 | H | 176 | 176 | 204 | 204 | 175 | 191 | 156 | 156 | 188 | 188 | 169 | 185 | 157 | 179 |
| 128 | Mar06 | H | 176 | 176 | 181 | 181 | 175 | 191 | 156 | 156 | 161 | 188 | 185 | 185 | 179 | 179 |
| 133 | Mar06 | H | 170 | 183 | 181 | 181 | 182 | 185 | 153 | 153 | 161 | 161 | 177 | 185 | 176 | 176 |
| 135 | Mar06 | H | 180 | 180 | 194 | 194 | 148 | 185 | 153 | 163 | 148 | 153 | 169 | 185 | 157 | 176 |
| 143 | Mar06 | H | 170 | 176 | 179 | 179 | 171 | 185 | 153 | 153 | 153 | 161 | 169 | 180 | 182 | 182 |
| 145 | Mar06 | H | 176 | 176 | 177 | 177 | 171 | 185 | 153 | 156 | 153 | 157 | 169 | 180 | 179 | 179 |
| 153 | Mar06 | H | 183 | 183 | 181 | 181 | 175 | 185 | 156 | 156 | 175 | 175 | 177 | 188 | 176 | 176 |
| 1022 | Aug06 | H | 176 | 176 | 181 | 181 | 171 | 171 | 156 | 156 | 153 | 153 | 185 | 185 | 179 | 185 |
| 1030 | Aug06 | H | 176 | 200 | 177 | 177 | 148 | 171 | 156 | 160 | 153 | 153 | 185 | 185 | 179 | 185 |
| 1032 | Aug06 | H | 170 | 170 | 181 | 181 | 185 | 185 | 156 | 160 | 161 | 161 | 185 | 185 | 157 | 176 |
| 1035 | Aug06 | H | 176 | 176 | 181 | 181 | 182 | 185 | 153 | 153 | 161 | 161 | 180 | 185 | 176 | 176 |
| 1039 | Aug06 | H | 170 | 180 | 177 | 177 | 175 | 182 | 156 | 160 | 157 | 161 | 175 | 185 | 179 | 182 |
| 1040 | Aug06 | H | 176 | 176 | 177 | 177 | 185 | 185 | 153 | 156 | 153 | 153 | 180 | 180 | 182 | 182 |
| 1061 | Aug06 | H | 176 | 176 | 181 | 181 | 191 | 191 | 156 | 156 | 153 | 161 | 169 | 185 | 179 | 179 |
| 1062 | Aug06 | H | 176 | 180 | 181 | 181 | 191 | 191 | 156 | 167 | 157 | 157 | 180 | 185 | 179 | 179 |
| 1064 | Aug06 | H | 180 | 200 | 177 | 181 | 163 | 171 | 156 | 156 | 148 | 148 | 180 | 180 | 179 | 185 |
| 1065 | Aug06 | H | 170 | 170 | 177 | 181 | 175 | 185 | 153 | 167 | 161 | 161 | 180 | 180 | 173 | 173 |
| 1071 | Aug06 | D | 180 | 180 | 177 | 181 | 175 | 191 | 153 | 153 | 157 | 165 | 169 | 180 | 179 | 182 |
| 1072 | Aug06 | H | 173 | 200 | 181 | 181 | 171 | 171 | 156 | 156 | 157 | 161 | 185 | 185 | 176 | 176 |
| 1091 | Aug06 | H | 176 | 183 | 181 | 204 | 185 | 185 | 156 | 156 | 153 | 153 | 180 | 180 | 182 | 185 |
| C10 | Aug06 | C | 180 | 180 | 181 | 181 | 175 | 191 | 153 | 153 | 153 | 161 | 185 | 185 | 179 | 182 |
| 2002 | Jan07 | D | 176 | 176 | 183 | 183 | 171 | 175 | 153 | 153 | 157 | 165 | 180 | 180 | 179 | 185 |
| 2007 | Jan07 | D | 178 | 178 | 181 | 181 | 171 | 185 | 156 | 156 | 150 | 188 | 169 | 169 | 179 | 182 |
| 2010 | Jan07 | H | 180 | 180 | 181 | 191 | 200 | 200 | 156 | 156 | 150 | 150 | 173 | 175 | 176 | 182 |
| 2016 | Jan07 | H | 180 | 180 | 177 | 177 | 175 | 175 | 156 | 156 | 153 | 153 | 180 | 180 | 176 | 176 |
| 2019 | Jan07 | D | 176 | 200 | 177 | 177 | 148 | 171 | 156 | 160 | 157 | 157 | 185 | 185 | 179 | 185 |
| 2020 | Jan07 | H | 180 | 180 | 177 | 177 | 171 | 175 | 156 | 156 | 153 | 153 | 185 | 185 | 182 | 195 |
| 2021 | Jan07 | H | 170 | 170 | 177 | 177 | 175 | 185 | 153 | 153 | 161 | 161 | 180 | 185 | 173 | 173 |
| 2022 | Jan07 | H | 176 | 176 | 177 | 177 | 148 | 171 | 160 | 160 | 153 | 153 | 185 | 185 | 179 | 185 |
| 2038 | Jan07 | H | 180 | 180 | 177 | 177 | 175 | 191 | 156 | 156 | 157 | 165 | 180 | 185 | 176 | 176 |
| 2040 | Jan07 | H | 176 | 176 | 177 | 177 | 171 | 185 | 156 | 156 | 150 | 150 | 185 | 188 | 176 | 179 |
| 2047 | Jan07 | H | 176 | 176 | 181 | 181 | 191 | 191 | 156 | 156 | 157 | 157 | 182 | 182 | 179 | 185 |
| 2049 | Jan07 | H | 183 | 183 | 185 | 185 | 148 | 148 | 156 | 156 | 157 | 165 | 185 | 185 | 176 | 182 |
| 2058 | Jan07 | H | 170 | 180 | 185 | 185 | 185 | 191 | 153 | 156 | 157 | 157 | 177 | 185 | 176 | 176 |
| 2059 | Jan07 | D | 173 | 180 | 181 | 181 | 171 | 175 | 156 | 156 | 153 | 153 | 185 | 188 | 179 | 195 |
| 2065 | Jan07 | H | 176 | 176 | 183 | 183 | 185 | 191 | 156 | 156 | 161 | 161 | 169 | 173 | 176 | 179 |
| 2072 | Jan07 | H | 173 | 173 | 189 | 189 | 185 | 191 | 153 | 153 | 157 | 165 | 175 | 180 | 176 | 176 |
| 2073 | Jan07 | H | 170 | 170 | 181 | 181 | 185 | 185 | 153 | 153 | 161 | 161 | 185 | 185 | 157 | 176 |
| 2079 | Jan07 | H | 170 | 170 | 179 | 179 | 175 | 175 | 156 | 156 | 161 | 170 | 185 | 188 | 179 | 182 |
| 2081 | Jan07 | D | 176 | 176 | 177 | 177 | 171 | 171 | 156 | 156 | 157 | 175 | 180 | 185 | 179 | 179 |
| 3001 | Jan07 | C | 180 | 180 | 183 | 183 | 185 | 185 | 156 | 156 | 178 | 178 | 185 | 185 | 176 | 176 |
| 3002 | Jan07 | C | 173 | 180 | 177 | 191 | 171 | 171 | 153 | 153 | 157 | 165 | 175 | 185 | 179 | 179 |
| 3003 | Jan07 | C | 180 | 180 | 181 | 181 | 185 | 185 | 156 | 156 | 175 | 175 | 169 | 180 | 195 | 195 |
| 3004 | Jan07 | C | 180 | 180 | 191 | 191 | 171 | 171 | 153 | 153 | 157 | 165 | 188 | 188 | 176 | 179 |
| 3005 | Jan07 | C | 170 | 170 | 179 | 179 | 175 | 185 | 153 | 153 | 161 | 161 | 180 | 180 | 173 | 192 |
| 3006 | Jan07 | C | 170 | 180 | 177 | 177 | 171 | 175 | 156 | 156 | 161 | 161 | 185 | 185 | 179 | 179 |
| 3007 | Jan07 | C | 176 | 176 | 177 | 177 | 148 | 171 | 160 | 160 | 157 | 165 | 185 | 185 | 179 | 179 |
| 3008 | Jan07 | C | 176 | 176 | 177 | 177 | 171 | 171 | 156 | 156 | 157 | 165 | 185 | 185 | 179 | 179 |
| 3009 | Jan07 | C | 176 | 176 | 177 | 177 | 148 | 171 | 156 | 160 | 153 | 153 | 169 | 185 | 179 | 179 |
| 3010 | Jan07 | C | 176 | 176 | 177 | 177 | 171 | 171 | 156 | 156 | 157 | 157 | 180 | 185 | 179 | 179 |
| 3011 | Jan07 | C | 176 | 176 | 177 | 177 | 171 | 171 | 156 | 156 | 157 | 165 | 185 | 185 | 179 | 179 |
| 3012 | Jan07 | C | 170 | 180 | 191 | 191 | 171 | 171 | 153 | 153 | 157 | 165 | 175 | 188 | 176 | 179 |
| 3013 | Jan07 | C | 176 | 176 | 181 | 181 | 175 | 175 | 160 | 160 | 163 | 165 | 180 | 180 | 176 | 179 |
| 3014 | Jan07 | C | 180 | 180 | 181 | 181 | 175 | 185 | 153 | 156 | 175 | 175 | 169 | 180 | 176 | 176 |
| 3015 | Jan07 | C | 176 | 176 | 177 | 177 | 171 | 171 | 156 | 156 | 157 | 175 | 180 | 185 | 179 | 182 |
| 3016 | Jan07 | C | 180 | 183 | 177 | 181 | 171 | 175 | 156 | 156 | 148 | 148 | 185 | 185 | 182 | 195 |
| 3017 | Jan07 | C | 176 | 178 | 181 | 181 | 171 | 191 | 156 | 156 | 153 | 153 | 169 | 185 | 179 | 179 |
| 3018 | Jan07 | C | 180 | 180 | 177 | 181 | 171 | 175 | 156 | 156 | 157 | 165 | 185 | 185 | 182 | 195 |
| 3019 | Jan07 | C | 180 | 180 | 181 | 185 | 178 | 178 | 156 | 156 | 157 | 165 | 185 | 185 | 195 | 195 |
| 3020 | Jan07 | C | 170 | 170 | 181 | 181 | 175 | 175 | 156 | 156 | 157 | 165 | 169 | 177 | 182 | 182 |
| 3021 | Jan07 | H | 176 | 176 | 177 | 177 | 171 | 171 | 156 | 156 | 157 | 157 | 180 | 185 | 179 | 179 |
| 3022 | Jan07 | H | 170 | 170 | 183 | 183 | 175 | 185 | 153 | 153 | 161 | 161 | 180 | 180 | 173 | 192 |
| 3033 | Jan07 | H | 170 | 170 | 179 | 179 | 175 | 175 | 153 | 156 | 161 | 161 | 177 | 177 | 173 | 192 |
| 3034 | Jan07 | H | 176 | 176 | 185 | 185 | 191 | 191 | 156 | 156 | 165 | 165 | 185 | 185 | 176 | 176 |
| *129* | *Mar06* | *H* | *180* | *180* | *183* | *183* | *171* | *171* | *156* | *156* | *157* | *157* | *180* | *185* | *-* | *-* |
| *130* | *Mar06* | *H* | *180* | *180* | *177* | *177* | *171* | *171* | *153* | *153* | *157* | *165* | *180* | *180* | *-* | *-* |
| *1048* | *Aug06* | *H* | *176* | *176* | *177* | *177* | *171* | *185* | *156* | *156* | *157* | *161* | *180* | *180* | *-* | *-* |
| *1085* | *Aug06* | *D* | *170* | *170* | *-* | *-* | *191* | *191* | *153* | *153* | *153* | *161* | *180* | *180* | *179* | *179* |
| *H21* | *Jan07* | *H* | *-* | *-* | *177* | *177* | *-* | *-* | *156* | *163* | *161* | *161* | *169* | *185* | *-* | *-* |
| *42* | *Mar06* | *H* | *-* | *-* | *181* | *181* | *-* | *-* | *163* | *163* | *-* | *-* | *-* | *-* | *-* | *-* |
| *115* | *Mar06* | *H* | *-* | *-* | *-* | *-* | *171* | *171* | *-* | *-* | *157* | *157* | *-* | *-* | *-* | *-* |
| *150* | *Mar06* | *H* | *-* | *-* | *-* | *-* | *171* | *171* | *-* | *-* | *157* | *157* | *-* | *-* | *-* | *-* |
| *1080* | *Aug06* | *D* | *-* | *-* | *-* | *-* | *-* | *-* | *156* | *156* | *153* | *153* | *-* | *-* | *-* | *-* |
| *1204* | *Aug06* | *H* | *-* | *-* | *-* | *-* | *171* | *171* | *-* | *-* | *153* | *153* | *-* | *-* | *-* | *-* |
| *2023* | *Jan07* | *H* | *-* | *-* | *-* | *-* | *185* | *185* | *156* | *156* | *-* | *-* | *-* | *-* | *-* | *-* |
| *41* | *Mar06* | *D* | *-* | *-* | *-* | *-* | *191* | *191* | *-* | *-* | *-* | *-* | *-* | *-* | *-* | *-* |
| *53* | *Mar06* | *D* | *-* | *-* | *183* | *183* | *-* | *-* | *-* | *-* | *-* | *-* | *-* | *-* | *-* | *-* |
| *63* | *Mar06* | *H* | *-* | *-* | *-* | *-* | *-* | *-* | *-* | *-* | *153* | *153* | *-* | *-* | *-* | *-* |
| *65* | *Mar06* | *D* | *-* | *-* | *-* | *-* | *-* | *-* | *-* | *-* | *153* | *153* | *-* | *-* | *-* | *-* |
| *84* | *Mar06* | *H* | *-* | *-* | *-* | *-* | *171* | *185* | *-* | *-* | *-* | *-* | *-* | *-* | *-* | *-* |
| *1014* | *Aug06* | *H* | *-* | *-* | *181* | *181* | *-* | *-* | *-* | *-* | *-* | *-* | *-* | *-* | *-* | *-* |
| *1059* | *Aug06* | *H* | *-* | *-* | *177* | *177* | *-* | *-* | *-* | *-* | *-* | *-* | *-* | *-* | *-* | *-* |
| *1076* | *Aug06* | *D* | *-* | *-* | *-* | *-* | *185* | *185* | *-* | *-* | *-* | *-* | *-* | *-* | *-* | *-* |
| *1099* | *Aug06* | *D* | *-* | *-* | *-* | *-* | *-* | *-* | *156* | *156* | *-* | *-* | *-* | *-* | *-* | *-* |
| *2005* | *Jan07* | *H* | *-* | *-* | *-* | *-* | *171* | *171* | *-* | *-* | *-* | *-* | *-* | *-* | *-* | *-* |
| *2036* | *Jan07* | *H* | *-* | *-* | *-* | *-* | *-* | *-* | *-* | *-* | *150* | *150* | *-* | *-* | *-* | *-* |
| *2055* | *Jan07* | *H* | *-* | *-* | *-* | *-* | *-* | *-* | *-* | *-* | *150* | *150* | *-* | *-* | *-* | *-* |
| *23593* | *Jan07* | *C* | *-* | *-* | *-* | *-* | *-* | *-* | *-* | *-* | *-* | *-* | *188* | *188* | *-* | *-* |
| *51* | *Mar06* | *D* | *-* | *-* | *-* | *-* | *-* | *-* | *-* | *-* | *-* | *-* | *-* | *-* | *-* | *-* |
| *68* | *Mar06* | *D* | *-* | *-* | *-* | *-* | *-* | *-* | *-* | *-* | *-* | *-* | *-* | *-* | *-* | *-* |
| *69* | *Mar06* | *D* | *-* | *-* | *-* | *-* | *-* | *-* | *-* | *-* | *-* | *-* | *-* | *-* | *-* | *-* |
| *72* | *Mar06* | *D* | *-* | *-* | *-* | *-* | *-* | *-* | *-* | *-* | *-* | *-* | *-* | *-* | *-* | *-* |
| *102* | *Mar06* | *H* | *-* | *-* | *-* | *-* | *-* | *-* | *-* | *-* | *-* | *-* | *-* | *-* | *-* | *-* |
| *121* | *Mar06* | *H* | *-* | *-* | *-* | *-* | *-* | *-* | *-* | *-* | *-* | *-* | *-* | *-* | *-* | *-* |
| *1021* | *Aug06* | *D* | *-* | *-* | *-* | *-* | *-* | *-* | *-* | *-* | *-* | *-* | *-* | *-* | *-* | *-* |
| *1050* | *Aug06* | *H* | *-* | *-* | *-* | *-* | *-* | *-* | *-* | *-* | *-* | *-* | *-* | *-* | *-* | *-* |
| *1057* | *Aug06* | *H* | *-* | *-* | *-* | *-* | *-* | *-* | *-* | *-* | *-* | *-* | *-* | *-* | *-* | *-* |
| *1060* | *Aug06* | *H* | *-* | *-* | *-* | *-* | *-* | *-* | *-* | *-* | *-* | *-* | *-* | *-* | *-* | *-* |
| *1067* | *Aug06* | *H* | *-* | *-* | *-* | *-* | *-* | *-* | *-* | *-* | *-* | *-* | *-* | *-* | *-* | *-* |
| *1081* | *Aug06* | *H* | *-* | *-* | *-* | *-* | *-* | *-* | *-* | *-* | *-* | *-* | *-* | *-* | *-* | *-* |
| *1082* | *Aug06* | *D* | *-* | *-* | *-* | *-* | *-* | *-* | *-* | *-* | *-* | *-* | *-* | *-* | *-* | *-* |
| *1086* | *Aug06* | *D* | *-* | *-* | *-* | *-* | *-* | *-* | *-* | *-* | *-* | *-* | *-* | *-* | *-* | *-* |
| *2004* | *Jan07* | *D* | *-* | *-* | *-* | *-* | *-* | *-* | *-* | *-* | *-* | *-* | *-* | *-* | *-* | *-* |
| *2039* | *Jan07* | *H* | *-* | *-* | *-* | *-* | *-* | *-* | *-* | *-* | *-* | *-* | *-* | *-* | *-* | *-* |
| *2053* | *Jan07* | *H* | *-* | *-* | *-* | *-* | *-* | *-* | *-* | *-* | *-* | *-* | *-* | *-* | *-* | *-* |
| *2082* | *Jan07* | *H* | *-* | *-* | *-* | *-* | *-* | *-* | *-* | *-* | *-* | *-* | *-* | *-* | *-* | *-* |
| *24520* | *Jan07* | *C* | *-* | *-* | *-* | *-* | *-* | *-* | *-* | *-* | *-* | *-* | *-* | *-* | *-* | *-* |
| *25529* | *Jan07* | *C* | *-* | *-* | *-* | *-* | *-* | *-* | *-* | *-* | *-* | *-* | *-* | *-* | *-* | *-* |
| *22580* | *Jan07* | *C* | *-* | *-* | *-* | *-* | *-* | *-* | *-* | *-* | *-* | *-* | *-* | *-* | *-* | *-* |
| *K9* | *Jan07* | *C* | *-* | *-* | *-* | *-* | *-* | *-* | *-* | *-* | *-* | *-* | *-* | *-* | *-* | *-* |
| *K19* | *Jan07* | *C* | *-* | *-* | *-* | *-* | *-* | *-* | *-* | *-* | *-* | *-* | *-* | *-* | *-* | *-* |
| *H1* | *Jan07* | *H* | *-* | *-* | *-* | *-* | *-* | *-* | *-* | *-* | *-* | *-* | *-* | *-* | *-* | *-* |
| *H8* | *Jan07* | *D* | *-* | *-* | *-* | *-* | *-* | *-* | *-* | *-* | *-* | *-* | *-* | *-* | *-* | *-* |

^*^ date of sampling; Mar06 = March 2006, Aug06 = August 2006, Jan07 = January 2007; ^†^ Sp = species sampled from; H = horse, D = donkey, C = cow; ^‡^ numbers designate the size of each allele in base-pairs. Samples in italics failed to amplify for all 7 markers.
