# Supplementary material for: Discovery of Mating in the Major African Livestock Pathogen Trypanosoma congolense
Source: PLoS One. 2009 May 15;4(5):e5564. doi: 10.1371/journal.pone.0005564 (PMC2679202; doi:10.1371/journal.pone.0005564)
Supplement: Table S4 — Allele frequencies at 7 microsatellite markers for the Gambian T. congolense population. The upper, non-italicised data are the frequencies for the 84 samples that amplified for all 7 loci. Samples in italics are frequencies for all samples (n = 133), including those that did not amplify for all loci. (0.07 MB DOC) [file pone.0005564.s006.doc]

| **Allele** | **TCM**  **1** | **TCM**  **2** | **TCM**  **3** | **TCM**  **4** | **TCM**  **5** | **TCM**  **6** | **TCM**  **7** |
| --- | --- | --- | --- | --- | --- | --- | --- |
| **1** | 0.161 | 0.054 | 0.363 | 0.274 | 0.048 | 0.054 | 0.286 |
| **2** | 0.036 | 0.369 | 0.190 | 0.018 | 0.238 | 0.054 | 0.369 |
| **3** | 0.381 | 0.375 | 0.202 | 0.595 | 0.065 | 0.268 | 0.131 |
| **4** | 0.042 | 0.054 | 0.125 | 0.095 | 0.208 | 0.446 | 0.071 |
| **5** | 0.298 | 0.018 | 0.054 | 0.006 | 0.119 | 0.101 | 0.054 |
| **6** | - | - | 0.024 | - | 0.190 | 0.012 | 0.024 |
| **7** | - | 0.054 | 0.024 | - | 0.030 | 0.018 | 0.048 |
| **8** | 0.036 | - | 0.006 | 0.012 | 0.006 | 0.036 | 0.018 |
| **9** | 0.006 | 0.054 | - | - | 0.006 | 0.012 | - |
| **10** | 0.042 | 0.012 | - | - | 0.006 | - | - |
| **11** | - | 0.012 | - | - | 0.065 | - | - |
| **12** | - | - | 0.012 | - | 0.006 | - | - |
| **13** | - | - | - | - | 0.012 | - | - |
| ***1*** | *0.165* | *0.049* | *0.391* | *0.269* | *0.067* | *0.050* | *0.282* |
| ***2*** | *0.034* | *0.359* | *0.198* | *0.016* | *0.247* | *0.061* | *0.376* |
| ***3*** | *0.375* | *0.386* | *0.177* | *0.597* | *0.057* | *0.289* | *0.129* |
| ***4*** | *0.040* | *0.049* | *0.130* | *0.086* | *0.201* | *0.428* | *0.071* |
| ***5*** | *0.307* | *0.016* | *0.047* | *0.005* | *0.108* | *0.100* | *0.053* |
| ***6*** | *-* | *-* | *0.021* | *-* | *0.211* | *0.011* | *0.024* |
| ***7*** | *-* | *0.071* | *0.021* | *-* | *0.021* | *0.017* | *0.047* |
| ***8*** | *0.034* | *-* | *0.005* | *0.027* | *0.005* | *0.033* | *0.018* |
| ***9*** | *0.006* | *0.049* | *-* | *-* | *0.005* | *0.011* | *-* |
| ***10*** | *0.040* | *0.011* | *-* | *-* | *0.005* | *-* | *-* |
| ***11*** | *-* | *0.011* | *-* | *-* | *0.057* | *-* | *-* |
| ***12*** | *-* | *-* | *0.010* | *-* | *0.005* | *-* | *-* |
| ***13*** | *-* | *-* | *-* | *-* | *0.010* | *-* | *-* |
