# Supplementary material for: Discovery of Mating in the Major African Livestock Pathogen Trypanosoma congolense
Source: PLoS One. 2009 May 15;4(5):e5564. doi: 10.1371/journal.pone.0005564 (PMC2679202; doi:10.1371/journal.pone.0005564)
Supplement: Table S5 — Hardy-Weinberg analysis for T. congolense populations including all samples that amplified for 7 microsatellite markers, and samples from subpopulations as defined by STRUCTURE. * P-value; those not significant at the 0.05 level are shown in bold. (0.04 MB DOC) [file pone.0005564.s007.doc]

|  | |  | Locus |  |  |  |  |  |  |
| --- | --- | --- | --- | --- | --- | --- | --- | --- | --- |
| Population | | | TCM  1 | TCM  2 | TCM  3 | TCM  4 | TCM  5 | TCM  6 | TCM  7 |
| All | P-val^*^ | | 0 | 0 | 0 | 0 | 0 | 0 | 0 |
| n = 84 | S.E | | 0 | 0 | 0 | 0 | 0 | 0 | 0 |
| W | P-val | | 0.02 | >0.01 | 0 | >0.01 | >0.01 | **0.05** | 0.12 |
| n = 14 | S.E | | >0.01 | >0.01 | 0 | >0.01 | >0.01 | >0.01 | >0.01 |
| X | P-val | | 0 | >0.01 | 0.02 | 0 | 0 | 0.01 | 0.01 |
| n = 27 | S.E | | 0 | >0.01 | >0.01 | 0 | 0 | >0.01 | >0.01 |
| Y | P-val | | 0.02 | 0 | **0.68** | >0.01 | **0.05** | 0.04 | >0.01 |
| n = 17 | S.E | | >0.01 | 0 | 0.01 | >0.01 | 0.01 | >0.01 | >0.01 |
| Z | P-val | | 0 | 0 | **0.89** | 0.04 | 0 | **0.18** | **0.28** |
| n = 26 | S.E | | 0 | 0 | >0.01 | >0.01 | 0 | 0.01 | 0.01 |
