# Supplementary material for: Discovery of Mating in the Major African Livestock Pathogen Trypanosoma congolense
Source: PLoS One. 2009 May 15;4(5):e5564. doi: 10.1371/journal.pone.0005564 (PMC2679202; doi:10.1371/journal.pone.0005564)
Supplement: Table S6 — Linkage disequilibrium (bold type) between pairwise loci in T. congolense samples from The Gambia, analysed for all samples that amplified for 7 microsatellite markers and samples from subpopulations as defined by the STRUCTURE programme. (0.07 MB DOC) [file pone.0005564.s008.doc]

Table S6. Linkage disequilibrium (bold type) between pairwise loci in *T. congolense* samples from The Gambia, analysed for all samples that amplified for 7 microsatellite markers and samples from subpopulations as defined by the STRUCTURE programme.

|  |  | W | n=14 | X | n=27 | Y | n=17 | Z | n=26 | all | n=84 |
| --- | --- | --- | --- | --- | --- | --- | --- | --- | --- | --- | --- |
| Locus#1 | Locus#2 | P | S.E. | P | S.E. | P | S.E. | P | S.E. | P | S.E. |
| TCM1 | TCM2 | 0.857 | 0.015 | 0.601 | 0.024 | 0.861 | 0.008 | 0.738 | 0.011 | **0.001** | 0.001 |
| TCM1 | TCM3 | 0.446 | 0.027 | 0.080 | 0.017 | **0.007** | 0.002 | 0.200 | 0.014 | **0.005** | 0.004 |
| TCM2 | TCM3 | **0.019** | 0.006 | 0.733 | 0.018 | 0.172 | 0.011 | 0.301 | 0.008 | **0.000** | 0.000 |
| TCM1 | TCM4 | 0.630 | 0.016 | 0.965 | 0.008 | 0.774 | 0.012 | 0.315 | 0.029 | 0.223 | 0.036 |
| TCM2 | TCM4 | 0.430 | 0.010 | 0.576 | 0.022 | 0.769 | 0.008 | 0.069 | 0.007 | 0.442 | 0.036 |
| TCM3 | TCM4 | 0.059 | 0.006 | 0.510 | 0.025 | 0.352 | 0.013 | **0.002** | 0.001 | **0.001** | 0.001 |
| TCM1 | TCM5 | 0.709 | 0.018 | 0.711 | 0.027 | 0.244 | 0.020 | 0.687 | 0.024 | 0.094 | 0.025 |
| TCM2 | TCM5 | 0.419 | 0.016 | 0.200 | 0.016 | 0.785 | 0.011 | 0.609 | 0.012 | 0.281 | 0.039 |
| TCM3 | TCM5 | 0.100 | 0.011 | 0.176 | 0.021 | 0.184 | 0.013 | 0.529 | 0.018 | **0.002** | 0.001 |
| TCM4 | TCM5 | 0.194 | 0.009 | 0.462 | 0.032 | 0.494 | 0.015 | 0.816 | 0.019 | 0.287 | 0.034 |
| TCM1 | TCM6 | 0.814 | 0.021 | **0.024** | 0.007 | 0.394 | 0.025 | 0.849 | 0.020 | **0.023** | 0.011 |
| TCM2 | TCM6 | **0.018** | 0.009 | 0.911 | 0.009 | 0.617 | 0.017 | 0.439 | 0.011 | **0.000** | 0.001 |
| TCM3 | TCM6 | 0.397 | 0.026 | **0.019** | 0.007 | **0.001** | 0.001 | **0.002** | 0.001 | **0.000** | 0.001 |
| TCM4 | TCM6 | **0.023** | 0.006 | 0.847 | 0.016 | 0.773 | 0.014 | 0.227 | 0.019 | 0.387 | 0.040 |
| TCM5 | TCM6 | 0.699 | 0.020 | 0.453 | 0.028 | **0.039** | 0.008 | **0.005** | 0.002 | **0.026** | 0.010 |
| TCM1 | TCM7 | 0.964 | 0.005 | 0.243 | 0.032 | 0.293 | 0.014 | 0.257 | 0.017 | **0.000** | 0.001 |
| TCM2 | TCM7 | **0.000** | 0.001 | 0.087 | 0.013 | 0.420 | 0.011 | **0.015** | 0.001 | **0.000** | 0.001 |
| TCM3 | TCM7 | **0.007** | 0.002 | **0.001** | 0.001 | **0.038** | 0.006 | 0.304 | 0.011 | **0.000** | 0.001 |
| TCM4 | TCM7 | 0.283 | 0.009 | 0.176 | 0.023 | 0.880 | 0.008 | 0.185 | 0.013 | 0.750 | 0.028 |
| TCM5 | TCM7 | 0.805 | 0.011 | 0.547 | 0.034 | **0.044** | 0.005 | 0.329 | 0.018 | 0.191 | 0.030 |
| TCM6 | TCM7 | 0.079 | 0.013 | 0.185 | 0.026 | **0.042** | 0.008 | 0.433 | 0.016 | **0.019** | 0.009 |
