# Supplementary material for: Discovery of Mating in the Major African Livestock Pathogen Trypanosoma congolense
Source: PLoS One. 2009 May 15;4(5):e5564. doi: 10.1371/journal.pone.0005564 (PMC2679202; doi:10.1371/journal.pone.0005564)
Supplement: Supplementary file 8 [file pone.0005564.s002.doc]

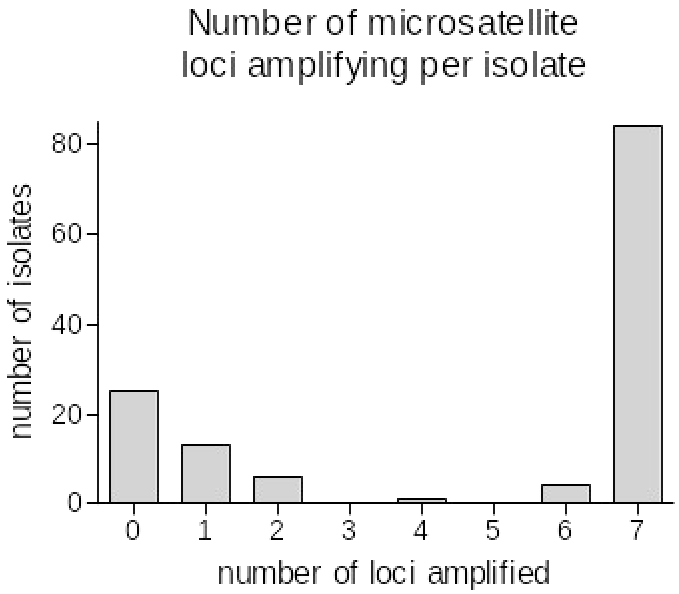
Number of microsatellite
loci amplifying per isolate
number of isolates
0
20
40
60
80
0
1
2
3
4
5
6
7
number of loci amplified
